# TRAUMATIC INJURIES OF THE CERVICAL SPINE: CURRENT EPIDEMIOLOGICAL PANORAMA

**DOI:** 10.1590/1413-785220182602185460

**Published:** 2018

**Authors:** MARCO AURÉLIO COTEGIPE NEGRELLI, RAFAEL GARCIA DE OLIVEIRA, IVAN DIAS DA ROCHA, ALEXANDRE FOGAÇA CRISTANTE, RAPHAEL MARTUS MARCON, TARCÍSIO ELOY PESSOA DE BARROS

**Affiliations:** 1. Spine Group, Instituto de Ortopedia e Traumatologia, Hospital das Clinicas HCFMUSP, Faculdade de Medicina, Universidade de São Paulo, São Paulo, SP, Brazil.; 2. Department of Orthopedics and Traumatology, Faculdade de Medicina, Universidade de São Paulo, São Paulo, SP, Brazil.

**Keywords:** Epidemiology, Spinal injuries, Cervical vertebrae/injuries., Epidemiologia, Traumatismos da coluna vertebral, Vértebras cervicais/lesões.

## Abstract

**Objective:**

To collect data from patients with cervical fracture who were treated surgically in a tertiary health service, in order to better understand the current scenario of this kind of injury in our population.

**Methods:**

This retrospective survey examined consecutive cases of patients with cervical spine trauma who received surgical treatment during 2013 and 2014. The data were subjected to descriptive statistical analysis.

**Results:**

Fifty-two patients were treated with surgery during 2013 and 2014. All patients classified as Frankel A and B developed respiratory failure. Patients classified as Frankel A, B, and C had significantly higher rates for postoperative complications (p < 0.01) than patients classified as Frankel D and E, except for the rate of postoperative infections (p = 0.717). Hospitalization time was also longer in the first group (p < 0.01).

**Conclusion:**

Patients with cervical trauma who present with neurological deficit at hospital admission should receive special attention, since the rate of postoperative complications is higher and hospital stays are lengthier in this group. In addition, patients with Frankel A and B classification should be monitored in an intensive care unit. Level of Evidence III; Retrospective comparative study.

## RESUMO

**Objetivo:**

Levantar dados de prontuário dos pacientes com fratura cervical submetidos a tratamento cirúrgico em um serviço de saúde terciário, para melhor compreensão do cenário atual desse tipo de lesão em nosso meio.

**Métodos:**

O estudo realizou o levantamento retrospectivo de casos consecutivos de pacientes com trauma da coluna cervical submetidos ao tratamento cirúrgico durante os anos de 2013 e 2014. Os dados foram submetidos à análise estatística descritiva.

**Resultados:**

Cinquenta e dois pacientes foram operados neste serviço entre os anos de 2013 e 2014. Todos os pacientes classificados como Frankel A e B evoluíram com insuficiência respiratória. Quando comparados, os pacientes Frankel A, B e C e Frankel D e E, a taxa de todos os tipos de complicações pós-operatórias foi maior no primeiro grupo, com significância estatística (p < 0,01), exceto a taxa de infecções pós-operatórias (p = 0,717). Além disso, o tempo de internação foi maior no primeiro grupo (p < 0,01).

**Conclusão:**

Pacientes com trauma cervical que se apresentem com déficit neurológico na admissão hospitalar devem receber especial atenção, uma vez que a taxa de complicações pós-operatórias e os dias de internação são maiores. Além disso, pacientes Frankel A e B devem ser monitorados em ambiente de terapia intensiva. Nível de Evidência III; Estudo retrospectivo comparativo.

## INTRODUCTION

Traumatic spine injuries are a severe public health problem, especially with respect to the context in which they occur and their severity potential. These injuries are associated with high-energy trauma such as auto accidents, and have a major impact in our field, sometimes leading to tragic outcomes such as irreversible spinal cord injury and death.^1^ ^,^ ^2^

The total frequency of spinal cord injury (SCI) is estimated from 27 to 47 per million in the entire population and approximately 6% in polytraumatized patients; 40% of this total may present some degree of neurological deficit, whether in the spinal cord lesion or injury to the nerve root.^1^ These data, however, are difficult to obtain due to the high association of SCI with fatal outcomes, which complicates the diagnosis.^3^

Injuries to neurological structures are more closely related to spinal trauma due to the spine’s anatomical characteristics, such as greater mobility and lower bone mass. The upper vertebrae, C1 and C2, have low correlation with SCI due to the greater diameter of the vertebral canal in this region.^4^

The most frequent trauma mechanisms are accidents (39-55%), causes related to urban violence (14-29%), falls (18-23%), and sports injuries (7-11%).^5^ A bimodal peak can be seen in the age distribution of traumatic spinal fractures and dislocations, which are more common in young adults (15-24 years) and middle-aged individuals (>55 years).^6^

The initial diagnosis is extremely important for proper patient management. A high rate of suspicion and care protocols for polytraumatized patients (Prehospital Trauma Life Support and Advanced Trauma Life Support*)* recommend maintaining a cervical collar until the presence of cervical injuries can be ruled out. Some authors advocate routine computerized tomographic (CT) scans in initial care provided to trauma patients to reduce the rates of diagnostic neglect.^7^

Several classifications have been created to categorize the various types of fracture in order to indicate precise treatment and allow comparison in different studies.^8^ The most common classifications used currently are the Subaxial Cervical Spine Injury Classification (SLIC) and the AO classification for cervical fractures.^9^ ^,^ ^10^

Intervention can be divided into two stages: fracture reduction and internal fixation. The literature is still controversial regarding the time and the ideal method for achieving reduction, which can be closed (with skull traction) or open (via anterior or posterior approaches). The closed procedure is not without risks, since cervical facet dislocations may be associated with traumatic disc hernias at the injury site in up to 54% of the cases, with the risk that these disc fragments could worsen the neurological injury by invading the vertebral canal during reduction.^11^ ^,^ ^12^ Some authors recommend nuclear magnetic resonance imaging (NMR) prior to installing skull traction to rule out the presence of hernia.^7^ ^,^ ^11^ ^,^ ^12^ However, NMR is not always easily available, and if the patient is awake and alert many physicians install the cranial halo in the urgent care ward without NMR, with the backing of more recent studies such as Vaccaro et al.,^7^ and obtain good results and low complication rates.^11^ ^,^ ^13^ ^,^ ^14^

Due to the scarcity of data in the Brazilian literature, especially with regard to epidemiological information on patients with cervical trauma (since much of the data is lost in pre-hospital care and many services do not record this information in a comprehensive manner), we decided to survey cases of cervical fracture and/or dislocation which were treated with surgery in our service in 2013 and 2014.

Therefore, the objective of this study is to collect data from the medical records of these patients to better understand the current scenario of traumatic cervical spine injuries in our area. This information can help improve actions in the areas of prevention, care, and management of patients with this type of injury.

## MATERIALS AND METHODS

This study was approved by the institutional review board (IOT-HCFMUSP process number 1186), as well as by the Plataforma Brasil ethics committee (70950417.4.0000.0068).

We performed a retrospective survey of the data contained in the records of consecutive patients with a diagnosis of cervical spine trauma who were treated and operated on in a tertiary-care hospital during 2013 and 2014, a total of 52 cases.

Epidemiological and preoperative data including clinical history, trauma mechanism, associated injuries, radiological classification, and severity of neurological injury were collected, along with surgical data such as access routes, surgical time, single or staged treatment, and need for intraoperative blood transfusion. Other data were related to patient hospitalization such as length of stay, neurological progress, death rate, and postoperative complications. The exclusion criteria were patients with cervical spine injuries of tumoral or infectious origin, patients treated without surgery, and pediatric patients (under 18 years of age).

The statistical analysis was performed using STATA, Statistics/Data Analysis software (Stata v14.2, StataCorp, College Station, Texas). For the categorical variables, the Mann-Whitney test was used. The normality of the continuous variables was accessed by the Shapiro-Wilk test, and the Mann-Whitney test was used for non-parametric data. The data collected were subjected to descriptive statistical analysis. Statistical significance was defined as *p*<0.05.

## RESULTS

**Figure 1:**
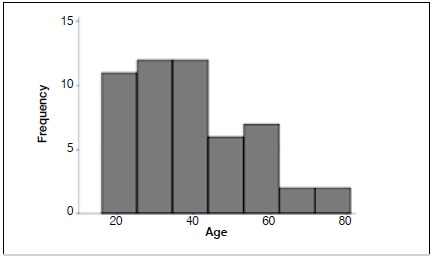
Frequency of fractures by age.

In 2013 and 2014, 52 cases of cervical trauma injuries were treated in our service. The sex ratio was 6.43:1 (45 men and 7 women). The mean age was 35.65 years (± 15.42), distributed with a bimodal peak of incidence, as shown in Figure 1.

**Figure 2:**
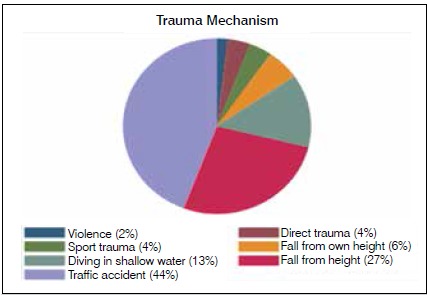
Trauma mechanism.

The most frequent mechanism of trauma was traffic accident, in 23 cases (44%), followed by falls from height in 14 cases (27%), and diving in shallow water in 7 cases (13%). Other observed trauma mechanisms were fall from own height, direct trauma, sports trauma, and violence, and the distribution of these mechanisms can be seen in Figure 2.

The cervical fractures were classified as high (affecting the first and second cervical vertebrae) or low (occurring between the third and seventh cervical vertebrae), with a frequency of 17% and 96%, respectively. In 8% of the total number of patients concomitant fractures in the high and low cervical spine were identified, and in three of the seven patients with odontoid fracture, associated subaxial fracture was also seen. When we isolated the low cervical fractures and classified them according to the AO classification for low cervical fractures, we found that type C fractures predominated, with 35 cases (70%).

In the 52 patients comprising our sample, 39 (75%) had isolated injuries in the cervical region, while 12% had concomitant fractures in the lower limbs, 8% in the upper limbs, 8% had cranioencephalic trauma, and 8% had fractures in other segments of the spine.

Most of the patients (63.46%) arrived at our service without neurological deficits, but 8 (15.38%) were initially assessed as Frankel grade D, 6 (11.54%) Frankel C, 4 (7.69%) Frankel B, and only 1 patient (1.92%), who was injured in an automobile accident and had a C4-C5 fracture/dislocation, arrived with a complete deficit. Table 1 correlates the initial Frankel score with type of fracture.

**Table 1:** Correlation between fracture type and Frankel score. Fractures (lines) were categorized as high (C1 - C2) and low (AO classification). Frankel score (columns).

Fracture Type vs. Frankel	A	B	C	D	E
High fracture	0	0	0	1	7
A	0	0	0	2	5
B	0	0	3	1	1
C	1	4	0	5	22

The median length of hospital stay was 17 days (interquartile range: 10 to 36 days). Table 2 correlates hospitalization time with the patient’s initial Frankel score.

**Table 2:** Days of hospitalization vs. initial Frankel score.

Initial Frankel	Mean	Frequency
A	136	1
B	83 (±24)	4
C	47 (±24)	6
D	33 (±38)	8
E	17 (±11)	33
Total	29	52

Another finding related to Frankel score is that when patients classified as Frankel A, B, and C (Group 1) were compared with Frankel D and E patients (Group 2), postoperative complications were more frequent in the first group, at a statistically significant level (*p*<0.01), with the exception of postoperative infection (*p*=0.717). In addition, hospitalization time was significantly greater in Group 1 (*P*<0.001), as shown in Table 3. Another relevant fact is that all five patients who were initially assessed as Frankel grade A or B developed respiratory failure during the course of hospitalization and required ventilator support.

**Table 3:** Comparison of complications among groups - Group 1 (Frankel A, B, and C), Group 2 (Frankel D and E). P value calculated by Fisher’s exact test for categorical variables and the Mann-Whitney test for the time of hospitalization (normal distribution of data was not observed according to the Shapiro-Wilk test).

	Group 1	Group 2	p
Total number of patients	11	41	-
Pressure ulcer	6	2	0.001
Acute kidney failure	5	3	0.007
Pneumonia	4	1	0.005
Urinary tract Infection	7	3	<0.001
Postoperative infection	1	4	0.717
Death	3	0	0.007
Time of hospitalization - median, in days	76	15	0.001
Interquartile interval	39-110	10-21	-

Half of the patients were placed into skeletal traction with a cranial halo in the emergency room, and bloodless reduction was achieved in 42.31% of cases. Complications were not seen in any patients during installation of the cranial halo, and neurological deficit did not worsen in any patient after the procedure. Only one patient presented nystagmus during traction and the procedure was interrupted, which caused immediate improvement of this symptom.

**Figure 3:**
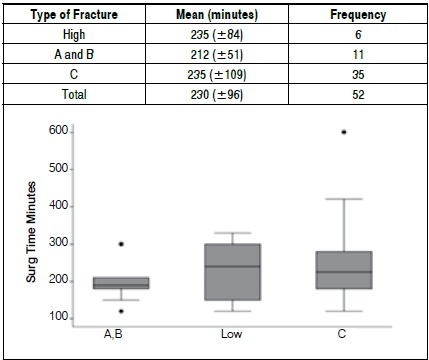
Mean surgical time vs. type of fracture (low fractures were classified by AO classification and type A and B were grouped together).

The average time of surgery for the patients included in the study was 230 minutes (±96) (Figure 3). Only three patients (5.7%) required blood transfusion during surgery, but in about 35% of the cases patients went to the intensive care unit (ICU) after surgery, in accordance with criteria defined by the anesthesia team. The surgical route selected for treatment was the anterior approach in 29 patients, posterior in 20 patients, and a combination of both routes in 3 patients.

Of the 19 patients who presented some degree of neurological deficit in the initial evaluation, 10 (52%) improved at least one Frankel grade during follow-up. Of the 52 patients, 6 (11.5%) required additional surgery: 5 (9.6%) to treat postoperative infection, requiring surgical debridement combined with antibiotic therapy, and 2 (3.8%) to extend the arthrodesis. Of the total number of patients, 3 (5.7%) died during hospitalization; all of these had neurological deficit when they initially arrived for treatment.

## DISCUSSION

As observed by Kraus et al.,^6^ we found a bimodal age distribution in fracture cases (Figure 1); the first and more significant peak occurred between 20 and 35 years of age, and the second peak at around 55 years of age. Fractures predominated significantly in males (86%).

The most frequent mechanism of trauma was traffic accidents (44%), followed by falls from height (27%), and diving in shallow water (13%). Together, these mechanisms corresponded to 84% of the cases in this study, highlighting the need for public policies to reduce car accidents, guidance about the risk of falls from height in the workplace, and warnings about the risks of diving in shallow water in order to prevent these incidents and possibly reduce the costs of healthcare directed toward victims of these accidents.^15^ ^,^ ^16^

Analysis of the frequency of neurological deficit in patients with high cervical fractures showed that only one was classified as Frankel D among the nine individuals with such fractures, while the other eight did not present any kind of deficit. This finding is consistent with the literature and can be explained by the difference in the size of the medullary canal between the low and high cervical spine.^16^

It is important to emphasize that all patients included in this study who were classified as Frankel A and B developed respiratory failure and required ventilator support. In a systematic review, Berney et al.^17^ showed that in patients with neurological deficits, the rate of respiratory complications was 84% in C1 to C4 fractures and 60% in C5 to T1 fractures, with tracheal intubation required in approximately 74% of cases. The findings from this present study as well as the literature therefore recommend that patients with traumatic cervical spine injuries and severe neurological deficits (Frankel A or B) by monitored in the ICU after initial stabilization upon arrival, in order to maintain control of respiratory function and respond quickly if ventilatory function worsens.^17^ ^,^ ^18^

As shown in Table 3, patients with more severe neurological deficits (Frankel A, B and C) had significantly greater complications and considerably longer hospitalizations than the other group (Frankel D and E). Consequently, health services that provide care for patients with cervical injuries should be prepared for more prolonged hospitalizations and more complications in patients with more severe deficits at the initial evaluation.

Our service has broad experience in initial treatment of cervical fracture/dislocation with the cranial halo, when this is indicated. In many cases, from an institutional point of view it is not possible to provide definitive, urgent surgical treatment for these patients. In these situations, the cranial halo plays an important role in reestablishing the diameter of the spinal canal, providing greater control of neurological prognosis.

In our study, 26 patients received a cranial halo, 23 with AO type C cervical injuries and 3 with the odontoid fractures. In this group of patients there was only one complication related to the cranial halo: one patient developed nystagmus upon reaching 13 kg of traction. At that moment, we chose to gradually reduce the traction, until 6 kg was reached and symptoms resolved completely. This example shows the importance of continuous monitoring by the medical team during weight increase in skeletal traction, through serial examinations and continuous evaluation of vital signs.

Our current practice is to increase traction by 1 kg/hour until reduction of the vertebral dislocation is achieved, or the maximum weight is reached (an initial 5 kg + 2.3 kg per vertebra level up to the level of injury), performing an X-ray each time after weight is added; this is slightly more conservative than the recommendation by Brun et al.^19^ to add 1 kg each 30 minutes, but we believe this practice allows patients to better adapt to the progressive increase in weight.

Of the 7 patients with odontoid fractures, 3 (42%) had associated subaxial cervical fractures. This data differs from Burke et al.,^20^ who found this combination in only 8% of patients. Among other factors, this difference can be explained by continuous improvements in the quality and definition of diagnostic methods (which identify fractures that previously went unnoticed), the significant difference in sample sizes between our two studies, and finally by the fact that this present study only analyzed cases treated surgically, which tend to result from trauma with greater energy.

## CONCLUSIONS

Patients with cervical trauma who present with neurological deficits at hospital admission should receive special attention, since their rates of postoperative complications and hospital stays are significantly greater than for other patients. Furthermore, patients classified as Frankel A and B should be monitored in an ICU because their risk of developing respiratory failure during hospitalization.
